# Inflammatory markers in the emergency department and PTSD symptoms in the AURORA Study: A longitudinal cohort study

**DOI:** 10.1017/S0033291726103833

**Published:** 2026-05-08

**Authors:** Kristen Nishimi, Sarah D. Linnstaedt, Thomas C. Neylan, Lauren A. McKibben, Liz Marie Albertorio-Sáez, Ying Zhao, Stacey L. House, Francesca L. Beaudoin, Xinming An, Jennifer S. Stevens, Gari D. Clifford, Tanja Jovanovic, Laura T. Germine, Scott L. Rauch, John P. Haran, Alan B. Storrow, Paul I. Musey, Phyllis L. Hendry, Sophia Sheikh, Brittany E. Punches, Robert A. Swor, Jose L. Pascual, Mark J. Seamon, Elizabeth M. Datner, Claire Pearson, David A. Peak, Roland C. Merchant, Robert M. Domeier, Niels K. Rathlev, Brian J. O’Neil, Paulina Sergot, Leon D. Sanchez, Steven E. Bruce, Steven E. Harte, Ronald C. Kessler, Karestan C. Koenen, Kerry J. Ressler, Samuel A. McLean, Aoife O’Donovan

**Affiliations:** 1 Mental Health, San Francisco VA Health Care System, USA; 2 Department of Psychiatry and Behavioral Sciences, University of California San Francisco, USA; 3 Institute for Trauma Recovery, UNC-Chapel Hill: The University of North Carolina at Chapel Hill, USA; 4 Department of Anesthesiology, UNC-Chapel Hill: The University of North Carolina at Chapel Hill, USA; 5 Departments of Psychiatry and Neurology, University of California San Francisco, USA; 6 Department of Emergency Medicine, Washington University School of Medicine in Saint Louis: Washington University, USA; 7 Department of Epidemiology, Brown University, USA; 8 Department of Emergency Medicine, Brown University, USA; 9 Department of Psychiatry and Behavioral Sciences, Emory University School of Medicine, USA; 10 Department of Biomedical Informatics, Emory University School of Medicine, USA; 11 Department of Biomedical Engineering, Georgia Institute of Technology and Emory University, USA; 12 Department of Psychiatry and Behavioral Neurosciences, Wayne State University, USA; 13 Institute for Technology in Psychiatry, McLean Hospital, USA; 14 The Many Brains Project, USA; 15 Department of Psychiatry, Harvard Medical School, USA; 16 Department of Psychiatry, McLean Hospital, USA; 17 Department of Emergency Medicine, University of Massachusetts Chan Medical School, USA; 18 Department of Emergency Medicine, Vanderbilt University Medical Center, USA; 19 Department of Emergency Medicine, Indiana University School of Medicine, USA; 20 Department of Emergency Medicine, University of Florida College of Medicine - Jacksonville, USA; 21 Department of Emergency Medicine, Ohio State University College of Medicine, USA; 22 Ohio State University College of Nursing, USA; 23 Department of Emergency Medicine, Oakland University William Beaumont School of Medicine, USA; 24 Departments of Surgery and Neurosurgery, University of Pennsylvania, USA; 25 Perelman School of Medicine, University of Pennsylvania, USA; 26 Department of Surgery, Division of Traumatology, Surgical Critical Care and Emergency Surgery, University of Pennsylvania, USA; 27 Department of Emergency Medicine, Jefferson Einstein Hospital, Jefferson Health, USA; 28 Department of Emergency Medicine, Sidney Kimmel Medical College, Thomas Jefferson University, USA; 29 Department of Emergency Medicine, Wayne State University, Ascension St. John Hospital, USA; 30 Department of Emergency Medicine, Massachusetts General Hospital, USA; 31 Department of Emergency Medicine, Harvard Medical School, USA; 32 Department of Emergency Medicine, Brigham and Women’s Hospital, USA; 33 Department of Emergency Medicine, Trinity Health-Ann Arbor, USA; 34 Department of Emergency Medicine, University of Massachusetts Chan Medical School - Baystate Campus: Baystate Medi, USA; 35 Department of Emergency Medicine, Wayne State University, Detroit Receiving Hospital, USA; 36 Department of Emergency Medicine, McGovern Medical School at UTHealth, USA; 37 Department of Psychological Sciences, University of Missouri–St. Louis, USA; 38 Department of Anesthesiology, University of Michigan Medical School, USA; 39 Department of Internal Medicine-Rheumatology, University of Michigan Medical School, USA; 40 Department of Health Care Policy, Harvard Medical School, USA; 41 Department of Epidemiology, Harvard T.H. Chan School of Public Health, Harvard University, USA; 42 Division of Depression and Anxiety, McLean Hospital, USA; 43 Department of Emergency Medicine, UNC-Chapel Hill: University of North Carolina at Chapel Hill, USA; 44 Department of Psychiatry, UNC-Chapel Hill: University of North Carolina at Chapel Hill, USA; 45 Weill Institute for Neurosciences, University of California San Francisco, USA

**Keywords:** cytokinesinflammationpost-traumatic stress disordertrauma

## Abstract

**Background:**

Systemic inflammation is hypothesized to contribute to post-traumatic stress disorder (PTSD) vulnerability. Few studies have examined inflammation shortly after trauma as a predictor of later PTSD symptoms. We examined whether inflammation from the emergency department (ED) post-trauma is associated with PTSD symptom severity over the following 6 months.

**Methods:**

Our sample included 742 AURORA participants, a longitudinal cohort of patients in 29 EDs across the United States after a traumatic stressor, followed up to 6 months. Plasma cytokines were assessed from a study blood draw in the ED: an inflammatory index (standardized sum of generally pro-inflammatory markers interleukin [IL]-6, IL-8, tumor necrosis factor alpha [TNF-α], interferon gamma [IFN-γ]), and generally anti-inflammatory IL-10. PTSD symptoms were self-reported at 2 weeks, 8 weeks, 3 months, and 6 months post-ED. Covariate-adjusted repeated-measures regressions estimated associations between inflammation and PTSD symptoms, overall and sex-stratified.

**Results:**

Among 742 participants (age *m* = 40.0 [13.7]; 479 [64.6%] female), PTSD symptoms were elevated then modestly decreased over follow-up. Higher ED inflammation was associated with higher PTSD symptoms across follow-up (standardized symptoms *β* = 0.05, 95% CI: 0.01–0.09), adjusted for potential confounders. Higher pro-inflammatory index levels and IL-6, IL-8, and TNF-α were associated with higher PTSD symptoms in males only, while higher IL-10 was associated with higher PTSD symptoms in females only.

**Conclusions:**

Pro-inflammatory levels shortly after traumatic stress are associated with heightened PTSD symptoms, particularly among males. Inflammatory markers may prove useful additions to prediction models for PTSD following trauma, with attention to sex differences.

## Introduction

Exposure to traumatic stressors can result in various adverse sequelae, including post-traumatic stress disorder (PTSD). However, only a minority of individuals who experience trauma will develop PTSD, which is defined by symptoms of intrusion, avoidance, negative cognition and mood, and arousal and reactivity in the aftermath of a traumatic event, causing significant distress for more than 1 month (American Psychiatric Association, 2013). Although psychosocial and demographic risk factors have been identified for PTSD (e.g. female sex, history of psychopathology, cumulative lifetime trauma, and trauma severity) (Tortella-Feliu et al., 2019), we lack precision in identifying individuals at the highest risk for developing PTSD symptoms. Biological factors assessed in the aftermath of traumatic stress may advance prediction models for PTSD, as current machine learning prediction models relying on demographic, behavioral, and cognitive factors have relatively limited predictive value (Horwitz et al., 2024; Ramos-Lima et al., 2020; Schultebraucks & Galatzer-Levy, 2019). Identifying biological factors that predict PTSD, particularly from the peritraumatic period, could greatly improve our ability to predict and ultimately intervene to prevent PTSD development.

Mounting evidence implicates dysregulated immune function, including innate immune activation and inflammation, in the development of PTSD (Felger et al., 2016; Hori & Kim, 2019; Katrinli et al., 2022). Immune activation is a dynamic response to various noxious stimuli, including psychological stress (Marsland et al., 2017). Following psychological stress, the body upregulates immune activity, with inflammatory cytokines regulating key aspects of this process (Marsland et al., 2017). Dysregulation in this response, and particularly prolonged elevated peripheral inflammation, may be implicated in the development and maintenance of PTSD symptoms (Hori & Kim, 2019). Peripheral inflammation may influence psychological symptoms via peripheral activation, impacting neuroinflammation (Hori & Kim, 2019). In particular, activated microglia and astrocytes in the brain may release pro-inflammatory cytokines that impact fear learning and memory processes, particularly by impacting functioning in threat and memory-related brain circuits, ultimately influencing the development of PTSD symptoms (Hori & Kim, 2019; Sah & Singewald, 2025). Additionally, continued low-grade systemic inflammation could impair fear extinction processes, contributing to maintenance and impeding resolution of PTSD symptoms (Bi et al., 2016; Quiñones et al., 2016). Related systems, like the hypothalamic–pituitary–adrenal axis and sympathetic nervous system (SNS), also respond to stress and can dynamically interact with the immune system to promote pro-inflammatory states (e.g. catecholamines released by the SNS mobilize monocytes and increase pro-inflammatory cytokine production) (Katrinli et al., 2022; Sah & Singewald, 2025). Moreover, elevated inflammation has the potential to directly elicit sleep disturbance, negative cognitions and emotions, and other symptoms of PTSD (Irwin, 2019; Jokela et al., 2016). Notably, our focus in the current work is the role of peripheral inflammation, but these other stress response systems and other psychophysiological processes likely also contribute to PTSD development (Mehta & Binder, 2012). Given that the blood–brain barrier is more permeable following stress (Welcome, 2020), the impact of peripheral inflammation on symptomology may be greatest shortly after trauma. Exact mechanisms are not fully delineated, and in particular, the relationship between peripheral and central inflammation and the temporal dynamics of these associations are complex (Bhatt et al., 2020; Bonomi et al., 2024; Hwang et al., 2025). However, we hypothesize that heightened peripheral inflammation, particularly during or immediately following traumatic stressors, could contribute to PTSD development and be a target for early intervention.

Chronic PTSD is characterized by elevated inflammation, as indexed by peripheral levels of inflammatory cytokines and other proteins (Ahmadian et al., 2020; Peruzzolo et al., 2022), though research examining prospective relationships between inflammation and later PTSD symptoms is mixed and has been limited in several ways. Small studies (*n*’s ≤ 100) have identified that higher peritraumatic blood levels of pro-inflammatory cytokines are associated with higher subsequent PTSD symptoms. Among 48 patients hospitalized with orthopedic injuries and 13 healthy gender-matched volunteers (mean age 39, 29% female), higher levels of the pro-inflammatory cytokine interleukin-8 (IL)-8 (but not IL-6) during hospitalization were associated with higher PTSS 1 month later (Cohen et al., 2011). In 70 patients in acute treatment for myocardial infarction (mean age 59, 20% female), higher scores on a pro-inflammatory index (plasma IL-1β, IL-6, and tumor necrosis factor alpha [TNF-α]) from within 2 days of admission were associated with higher levels of PTSD symptoms at 12 months, even adjusting for anti-inflammatory markers (IL-4, IL-10, and transforming growth factor-beta 1 [TGF-β1]) (von Känel et al., 2022). In contrast, higher levels of certain pro-inflammatory cytokines (TNF-α and interferon gamma [IFN-γ], but not IL-1β or IL-6) from the ED in the hours following various traumatic events were associated with *lower* risk for chronic PTSD symptoms over 1 year among 273 adults (mean age 36, 49% female) (Michopoulos et al., 2020). Follow-up analyses identified that this negative inflammation-PTSD symptom association was largely present in male compared to female individuals (Lalonde et al., 2021). Other studies have not identified associations between post-trauma inflammatory markers and PTSD. For example, comparing 53 individuals with mild traumatic brain injury and 24 controls with orthopedic injury assessed within a day of injury (mean age 27, 36% female), plasma IL-10 and IL-6 were not associated with PTSD symptoms 6 months later (Vedantam et al., 2021).

Existing empirical evidence has mostly smaller sample sizes, potentially limiting power, and varied methodologies that could have contributed to heterogeneity. There may be overestimation of associations between elevated inflammation and PTSD due to potential confounders; thus, it is critical to adjust for a range of sociodemographic, psychological, and health-related covariates (Bruenig et al., 2018; Nilsonne et al., 2016; Passos et al., 2016). Moreover, the value of biomarkers in PTSD prediction algorithms depends on their ability to predict over and above other variables. Finally, most evidence focuses on a few individual cytokines or inflammatory markers in isolation with PTSD. Inflammation is a complex, integrated, and dynamic response. Therefore, it is important to look at constellations of biomarkers as opposed to individual factors alone. Examining inflammatory index scores, which capture composite levels of inflammatory markers that reflect broad inflammatory activity and benefit from lower error variance than individual markers (Lindqvist et al., 2014; O’Donovan et al., 2013; van Dooren et al., 2016), may expand our understanding of how multiple physiological markers influence outcomes and improve prediction models.

In this study, we examined associations between inflammation in the ED after traumatic stress exposure and subsequent PTSD symptoms across 6 months in the Advancing Understanding of RecOvery afteR trauma (AURORA) Study, a large, diverse sample of individuals assessed shortly after exposure to trauma (McLean et al., 2020). We hypothesized that higher levels of an inflammatory index of peripheral plasma cytokines collected at the ED would be associated with higher levels of PTSD symptoms across 6 months of follow-up, given mixed prospective evidence combined with positive cross-sectional associations. To determine whether inflammation was associated with PTSD symptoms independent of other known risk factors and correlates, we adjusted for a range of sociodemographic, trauma, mental, and behavioral factors. Sex differences have been identified in immune functioning and peripheral inflammatory activity (Martínez de Toda et al., 2023; Oertelt-Prigione, 2012), PTSD risk and trajectories (Christiansen & Berke, 2020), and in the associations between inflammatory cytokines and PTSD symptoms (Lalonde et al., 2021). Therefore, we also explored potential sex differences.

## Subjects and methods

### Study design and participants

Data for this longitudinal cohort study came from the AURORA study (McLean et al., 2020). AURORA enrolled adults presenting to the ED in 29 hospitals across the United States following traumatic stressor exposure. Individuals were eligible if they were aged 18–75 years and presented to the ED within 72 hours of trauma exposure, including traumatic events like motor vehicle accidents or events involving actual or threatened serious injury, sexual violence, or death, either experienced, witnessed, or learned about. Individuals were excluded if they had general anesthesia, long bone fractures, significant hemorrhage, solid organ injury, were not alert or oriented, were pregnant or breastfeeding, experienced ongoing domestic violence, or had >20 mg morphine/day, among other study participation restrictions (see McLean et al., 2020). Eligible and consenting participants completed a variety of assessments (e.g. self-report, biological, and medical record review) in the ED and for up to 6 months post-ED. Of the 2943 individuals in the AURORA study, we restricted to those with at least one plasma inflammatory biomarker assayed at the ED and at least one PTSD symptom assessment across four subsequent time points over 6 months, resulting in an analytic sample of 742. Covariates were compared between the analytic sample and those in the broader AURORA cohort (Supplementary Table S1), suggesting the analytic sample was older, had lower income, more employment, lower area deprivation, greater pain medication use, lower tobacco use, a greater number of lifetime traumas, and more pre-trauma depression. The AURORA study was approved by each study site’s institutional review board. All participants provided written informed consent and were compensated for participation. The authors assert that all procedures contributing to this work comply with the ethical standards of the relevant national and institutional committees on human experimentation and with the Helsinki Declaration of 1975, as revised in 2008.

### Measures

Independent variables included plasma inflammatory cytokines from blood samples taken specifically for the AURORA study in the ED post-trauma. Inflammatory cytokines included IL-6, IL-8, TNF-α, IFN-γ, and IL-10 and were assayed in two batches with MesoScale Discovery assays (mean coefficients of variation: IL-6 = 4.1%, IL-8 = 3.0%, TNF-α = 5.4%, IFN-γ = 4.9%, and IL-10 = 8.1%). We examined potential batch effects, identifying that levels of TNF-α and IL-10, as well as distributions of covariates, differed by batch. As such, we adjusted for the cytokine assay batch (first vs. second). Given the positive skew of all inflammatory markers, we added 0.1 and log-transformed, then winsorized extreme values (> or < 3 SD of the mean) to that value (±3 SD of the mean) (Landau et al., 2019). An inflammatory index was created as the sum of standardized levels of log-transformed and winsorized IL-6, IL-8, TNF-α, and IFN-γ, all typically considered pro-inflammatory cytokines (Lindqvist et al., 2014; van Dooren et al., 2016).

Outcomes included PTSD symptoms self-reported by participants at 2 weeks, 8 weeks, 3 months, and 6 months post-ED. PTSD symptoms were assessed with the widely used and validated 20-item self-report PTSD Checklist for DSM-5 (PCL-5) (Frank W. Weathers et al., 2013). Severity of each symptom in the past month (past 2 weeks for 2 week timepoint) was reported on a scale of 0 (not at all) to 4 (extremely). Total symptom scores for each timepoint were derived, and probable PTSD was defined by a total symptom score ≥ 33, which has shown high sensitivity and specificity for probable PTSD diagnosis (Bovin et al., 2016). Internal consistency for total symptom scores was high at each time point (Cronbach’s alphas = 0.95–0.97). In secondary analyses, we calculated PTSD symptom cluster scores reflecting re-experiencing, avoidance, negative alterations in mood and cognition, and hyperarousal domains (Weathers, Litz, et al., 2013).

Covariates were assessed via self-report in the ED, 2-, or 8-week follow-up assessments. Measures included sociodemographic factors (i.e. sex assigned at birth, age, race/ethnicity [aggregated as Hispanic, Non-Hispanic Black, Non-Hispanic White, Non-Hispanic Other including Asian, Hawaiian, American Indian, or other race]), marital status, and socioeconomic status as indexed by education, annual family income, employment status, and state Area Deprivation Index (a measure of relative socioeconomic disadvantage). Additional relevant measures included the time from traumatic stressor exposure to blood draw, study site, and assay batch. Trauma and pre-trauma measures that could relate to both inflammation and PTSD symptoms post-trauma included trauma type, pain level in the ED (Abbreviated Injury Scale) (Loftis et al., 2018), the use of nonsteroidal anti-inflammatory drugs (NSAIDs) in the ED, childhood trauma (Childhood Trauma Questionnaire total score) (Bernstein et al., 2003), and lifetime trauma count (Life Events Checklist for DSM-5) (Weathers, Blake, et al., 2013). Pre-trauma measures also included retrospectively reported pre-ED PTSD symptoms assessed with an amended Brief PTSD Checklist (six items assessing disturbing memories, feeling upset, avoidance, feeling distant, feeling angry, and difficult concentrating rated from 0 to 4 [Cronbach’s α 0.82]; sum scores ≥8 were considered probable PTSD) (Lang & Stein, 2005) and pre-ED major depressive episode (MDE; PROMIS Depression measure items assessing diagnostic criteria for MDE in DSM-5, including ≥5 symptoms over 2 weeks, including depressed mood and/or loss of interest/pleasure) (Cella et al., 2010; Kessler & Ustün, 2004). Health and behavioral factors may reflect confounders, correlates, or pathway variables and include body mass index (BMI; derived as kg/m^2^ from self-reported weight and height), lifetime alcohol use (yes/no), and lifetime tobacco use (yes/no). To account for repeated PTSD symptom outcomes, time in weeks since the ED assessment at each follow-up time point was included in repeated-measures models.

### Statistical analysis

Our primary models estimated associations between the ED inflammatory index and repeated measures of PTSD symptoms across 6 months using repeated-measures linear regressions, which determine associations between ED inflammation and average PTSD symptom levels across time, including time since ED to PTSD assessment as a covariate. Inflammatory predictors and PTSD symptoms were standardized (*M* = 0, SD = 1) for analytic models, so estimates are interpreted as standardized *β*s. Time × inflammation interactions were estimated to determine whether associations between inflammation and PTSD symptoms varied across time (e.g. stronger associations earlier vs. later). Covariates were chosen a priori based on theoretical considerations and prior empirical evidence regarding their relevance to the association between inflammation and PTSD and were included in successive models adjusted for (1) time since ED; (2) time since ED, time from traumatic stressor exposure to blood draw, study site, assay batch, and sociodemographic factors as potential confounders; and (3) Model 2 plus trauma characteristics, pre-trauma exposures, pre-trauma mental health, health, and behavioral factors, which includes both potential confounders and potential moderators and mechanisms. Given the large number of related covariates, we examined variance inflation factors (VIFs) and pairwise correlations for covariates in Models 2 and 3 and did not identify evidence of multicollinearity (all corrected VIFs < 2, correlations < 0.20). Notably, while it is possible that acute inflammation was influenced by traumatic injury or acute psychological stress in the ED, and we adjust for some of these potential factors in Model 3, we are interested in levels of post-trauma inflammation agnostic to its causes.

There was some missingness of individual inflammatory markers, PTSD symptoms at a given time point, and covariates (see Table 1 and Supplementary Tables S2 and S3). In all analytic models, we used multiple imputation with chained equations (MICE; R *mice* package), which applies predictive mean matching for continuous variables and logistic regression for binary variables to impute 25 datasets for all missing variables (overall, 6.4% of data were imputed), and then calculated pooled estimates across datasets for each model.

**Table 1.** Baseline covariates among the analytic sample and their associations with the inflammatory index at the ED (*n* = 742)

Continuous covariates		Total	Inflammatory index	*p*-value
		*M* (SD)	r	
Age		37.99 (13.7)	0.17	0.76
State Area Deprivation Index		9.07 (3.4)	0.04	0.65
Time to blood draw, days		2.04 (13.7)	−0.02	0.48
ED pain level		6.38 (2.6)	0.12	0.95
BMI		31.41 (8.7)	0.24	0.47
Childhood trauma questionnaire		17.40 (15.7)	0.00	0.53
Lifetime trauma count		3.31 (2.8)	0.04	0.66

Categorical covariates		*N (%)*	*M* (SD)	*p*-value
Sex	Female	479 (64.6)	−0.07 (2.6)	0.26
	Male	263 (35.4)	0.15 (2.5)	
Race/ethnicity	Non-Hispanic White	255 (34.4)	0.03 (2.5)	0.20
	Non-Hispanic Black	382 (51.5)	−0.15 (2.5)	
	Hispanic	75 (10.1)	0.45 (2.6)	
	Non-Hispanic other	26 (3.5)	0.48 (3.2)	
Education	Less than high school	83 (11.2)	0.76 (2.7)	**0.01**
	High school graduate	180 (24.3)	0.11 (2.6)	
	Some college	205 (27.6)	−0.32 (2.5)	
	College graduate	273 (36.8)	−0.03 (2.4)	
Family income	<$19 k/month	236 (31.8)	−0.10 (2.5)	0.86
	$19–35 k/month	220 (29.6)	−0.05 (2.6)	
	More than $35 k/month	239 (32.2)	0.03 (2.5)	
Employment	Employed currently	514 (69.3)	−0.07 (2.5)	0.60
	Not employed currently	184 (24.8)	0.05 (2.6)	
Marital status	Married	149 (20.1)	0.14 (2.5)	0.62
	Previously married	141 (19.0)	0.10 (2.6)	
	Never married	447 (60.2)	−0.07 (2.6)	
Trauma type	Animal-related	23 (3.1)	1.30 (1.8)	**0.04**
	Burns	5 (0.7)	0.32 (3.4)	
	Falls, <10 feet	30 (4.0)	0.63 (2.8)	
	Falls, ≥10 feet	11 (1.5)	1.77 (3.0)	
	Incident with many people	2 (0.3)	−0.99 (1.1)	
	Motor vehicle collision	555 (74.8)	−0.09 (2.5)	
	Non-motorized collision	15 (2.0)	0.05 (3.0)	
	Physical assault	69 (9.3)	0.07 (2.3)	
	Sexual assault	4 (0.5)	−2.33 (1.7)	
	Other	28 (3.8)	−0.23 (2.8)	
ED NSAID use	Yes	254 (34.2)	−0.10 (2.6)	0.19
	No	349 (47.0)	0.18 (2.5)	
Alcohol use	Yes	437 (58.9)	0.03 (2.5)	0.76
	No	303 (40.8)	−0.02 (2.6)	
Tobacco use	Yes	235 (31.7)	0.07 (2.7)	0.67
	No	503 (67.8)	−0.02 (2.5)	
Pre-ED PTSD	PTSD	340 (45.8)	0.10 (2.6)	0.38
	No PTSD	402 (54.2)	−0.06 (2.5)	
Pre-ED MDE	MDE	172 (23.2)	−0.18 (2.6)	0.29
	No MDE	523 (70.5)	0.05 (2.5)	

*Note: P*-values are for correlations with continuous covariates or ANOVAs for mean levels of the inflammatory index across categorical covariates. Inflammatory index is the standardized sum of log-transformed and windsorized levels of IL-6, IL-8, TNF-α, and IFN-γ from plasma blood samples in the ED. Previously married includes separated, annulled, divorced, or widowed; BMI = body mass index, ED = emergency department, MDE = major depressive episode, NSAID = nonsteroidal anti-inflammatory drug, PTSD = post-traumatic stress disorder, SD = standard deviation. Missing: race/ethnicity *n* = 4 (0.5%), education *n* = 1 (0.1%), income *n* = 47 (6.3%), employment *n* = 44 (5.9%), marital status *n* = 5 (0.7%), state area deprivation index *n* = 5 (0.7%), time to blood draw *n* = 340 (45.8%), ED NSAID use *n* = 139 (18.7%), BMI *n* = 348 (46.9%), alcohol use *n* = 2 (0.3%), tobacco use *n* = 4 (0.5%), childhood trauma questionnaire *n* = 68 (9.2%), lifetime trauma count *n* = 16 (2.2%), pre-ED MDE *n* = 47 (6.3%).

A series of secondary analyses was conducted. We examined each inflammatory marker as a separate predictor of PTSD symptoms over time in repeated linear regression models. We tested inflammation × sex interactions in the primary models and performed subsequent sex-stratified models where potential sex differences in estimates were evident. To examine probable PTSD, we ran Poisson models with robust error variance to determine the relative risk (RR) of binary probable PTSD as a repeated outcome across follow-up. To determine whether inflammation was associated with dimensions of PTSD symptomology, we ran repeated linear regression models with individual PTSD symptom clusters over time as outcomes. Data were prepared and analyzed with R (version 4.4.0), and an *a priori* threshold of *p* < .05 was used for statistical significance.

## Results

### Sample characteristics

The sample of 742 individuals was 38 years old on average, including 64.6% women and 34.4% non-Hispanic White, 51.5% non-Hispanic Black, 10.1% Hispanic, and 3.5% other race individuals (Table 1). Inflammation levels were generally positively intercorrelated as expected, with bivariate cytokine correlations ranging between *r*s = 0.06–0.55 (Supplementary Table S2). Lower educational attainment was associated with higher inflammation, and inflammation varied across types of traumatic stressors (e.g. higher inflammation among those experiencing animal-related exposures or falls ≥10 feet) (Table 1). The inflammatory index did not significantly differ by sex, though IL-6 and IL-8 were marginally higher among males versus females (Supplementary Table S4).

PTSD symptoms decreased slightly over time from an average of 33.1 (SD = 19.8) at 2-weeks post-ED to 27.3 (SD = 21.1) at 6 months post-ED, with 42.7% and 33.2% meeting criteria for probable PTSD at both time points, respectively (Supplementary Table S3). Symptoms across time were modestly correlated (*r*s = 0.22–0.39). PTSD symptoms did not significantly differ by sex at 2- or 8-week post-ED, though females had higher symptoms at both 3- and 6-month post-ED (Supplementary Table S4).

### ED inflammation and PTSD symptoms over follow-up

**Figure 1.**
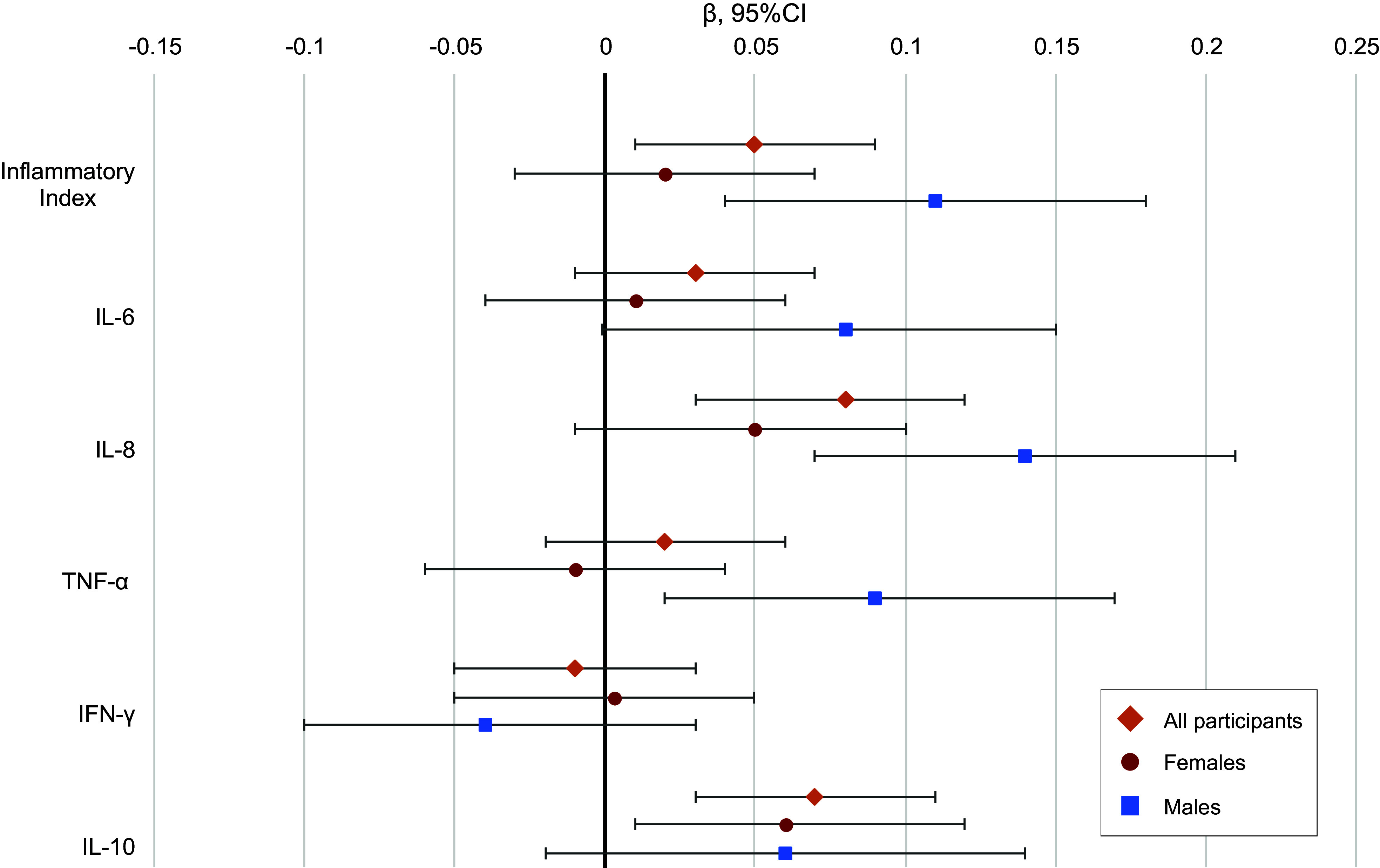
Overall and sex-stratified associations of inflammation with repeated PTSD symptoms over follow-up. *Note*: Estimates are standardized betas and 95% confidence interval bars for each inflammatory measure in the emergency department (ED) and repeated measures of PTSD symptoms over 6 months follow-up, adjusted for time since ED, age, race/ethnicity, education, income, employment, marital status, state area deprivation index, time from trauma to blood draw, site ID, and assay batch. Orange diamonds are all participants, red circles are female, blue squares are male.

Higher ED levels of the inflammatory index were associated with higher PTSD symptoms averaged across follow-up (Model 2 [confounder adjusted]: *β* = 0.05, 95% CI: 0.01, 0.09), with associations remaining consistent across increasingly adjusted models (Table 2, Figure 1; unadjusted associations plotted in Supplementary Figure S1). No inflammation × time interactions were significant (Supplementary Table S5), indicating that the magnitude of association between inflammation and PTSD symptoms was stable across time, and linear associations were determined as the best-fitting models (compared to quadratic).

**Table 2.** Associations between inflammatory markers and repeated measures of post-traumatic stress symptoms over 6 months (*n* = 742)

Predictor	Model 1			Model 2			Model 3		
	*β*	*95% CI*	*p*-value	*β*	*95% CI*	*p*-value	*β*	*95% CI*	*p*-value
Inflammatory index	**0.04**	**0.001, 0.08**	**0.047**	**0.05**	**0.01, 0.09**	**0.027**	**0.04**	**0.004, 0.08**	**0.031**
IL–6	0.03	−0.01, 0.06	0.202	0.03	−0.01, 0.07	0.111	*0.04*	*−0.001, 0.08*	*0.059*
IL–8	**0.07**	**0.03, 0.10**	**0.001**	**0.08**	**0.03, 0.12**	**<.001**	*0.04*	*−0.001, 0.08*	*0.057*
TNF-α	0.01	−0.02, 0.05	0.531	0.02	−0.02, 0.06	0.250	0.03	−0.02, 0.07	0.242
IFN-γ	−0.004	−0.04, 0.04	0.861	−0.01	−0.05, 0.03	0.635	0.01	−0.03, 0.05	0.682
IL–10	0.03	−0.01, 0.07	0.144	**0.07**	**0.03, 0.11**	**0.002**	**0.06**	**0.02, 0.11**	**0.005**

*Note:* Individual repeated-measures linear regressions; missingness addressed by multiple imputation and presenting pooled results from 25 datasets. Inflammatory index is standardized sum of log IL-6, IL-8, TNF-α, and IFN-γ. All inflammatory predictors and PTSD symptoms are standardized. Bold *p* < .05; *Italicized p < .10.* Model 1: time since ED. Model 2: time since ED, age, sex, race/ethnicity, education, income, employment, marital status, state area deprivation index, time from trauma to blood draw, site ID, and assay batch. Model 3: Model 1 and ED trauma type, ED pain level, ED NSAID use, childhood trauma questionnaire, lifetime trauma count, pre-ED PTSD, pre-ED depression, BMI, alcohol use, and tobacco use.

### Secondary analyses

When examining individual markers as predictors, higher levels of IL-8 (Model 2: *β* = 0.08, 95% CI: 0.03, 0.12) and IL-10 (Model 2: *β* = 0.07, 95% CI: 0.03, 0.11) were associated with higher PTSD symptoms (Table 2).

We found a marginally significant sex × inflammatory index interaction for PTSD symptoms suggestive of potential sex differences (Supplementary Table S5) and thus conducted sex-stratified models. Significant associations were identified between the inflammatory index and several individual markers and PTSD symptoms among males (Model 2 inflammatory index: *β* = 0.11, 95% CI: 0.04, 0.18; IL-8 *β* = 0.14, 95% CI: 0.08, 0.21; TNF-α *β* = 0.09, 95% CI: 0.02, 0.17), whereas only IL-10 was associated with PTSD symptoms in females (Model 2 IL-10: *β* = 0.06, 95% CI: 0.01, 0.12; Table 3 and Figure 1).

**Table 3.** Sex-stratified associations between inflammatory markers and repeated measures of post-traumatic stress symptoms over 6 months

Predictor	Model 1			Model 2			Model 3		
	*β*	*95% CI*	*p*-value	*β*	*95% CI*	*p*-value	*β*	*95% CI*	*p*-value
Female (*n* = 479)									
Inflammatory index	0.03	−0.02, 0.08	0.197	0.02	−0.03, 0.07	0.435	0.01	−0.04, 0.06	0.744
IL–6	0.004	−0.04, 0.05	0.863	0.01	−0.04, 0.06	0.758	0.01	−0.04, 0.06	0.726
IL–8	*0.04*	*−0.01, 0.09*	*0.078*	*0.05*	*−0.01, 0.10*	*0.083*	0.002	−0.05, 0.06	0.951
TNF-α	0.01	−0.03, 0.05	0.583	−0.01	−0.06, 0.04	0.786	−0.01	−0.06, 0.04	0.762
IFN-γ	0.02	−0.03, 0.07	0.454	0.003	−0.05, 0.05	0.897	0.01	−0.04, 0.06	0.582
IL–10	**0.05**	**0.003, 0.10**	**0.037**	**0.06**	**0.01, 0.12**	**0.019**	*0.05*	*−0.002, 0.11*	*0.058*
Male (*n* = 263)									
Inflammatory index	*0.06*	*−0.001, 0.13*	*0.055*	**0.11**	**0.04, 0.18**	**0.004**	**0.10**	**0.03, 0.17**	**0.008**
IL–6	**0.07**	**0.01, 0.14**	**0.033**	*0.08*	*−0.002, 0.15*	*0.056*	*0.07*	*−0.01, 0.15*	*0.075*
IL–8	**0.13**	**0.07, 0.19**	**<.001**	**0.14**	**0.08, 0.21**	**<.001**	**0.09**	**0.02, 0.16**	**0.013**
TNF-α	0.02	−0.05, 0.08	0.601	**0.09**	**0.02, 0.17**	**0.016**	**0.09**	**0.01, 0.18**	**0.026**
IFN-γ	*−0.06*	*−0.13, 0.001*	*0.052*	−0.03	−0.10, 0.04	0.342	−0.01	−0.08, 0.07	0.892
IL–10	0.002	−0.06, 0.07	0.963	0.06	−0.02, 0.14	0.125	0.04	−0.04, 0.12	0.276

*Note:* Individual repeated-measures linear regressions; missingness addressed by multiple imputation and presenting pooled results from 25 datasets. Inflammatory index is standardized sum of log IL-6, IL-8, TNF-α, and IFN-γ. All inflammatory predictors and PTSD symptoms are standardized. Bold *p* < .05; *Italicized p < .10.* Model 1: time since ED. Model 2: time since ED, age, race/ethnicity, education, income, employment, marital status, state area deprivation index, time from trauma to blood draw, site ID, and assay batch. Model 3: Model 1 and ED trauma type, ED pain level, ED NSAID use, childhood trauma questionnaire, lifetime trauma count, pre-ED PTSD, pre-ED depression, BMI, alcohol use, and tobacco use.

Patterns of results were similar when considering binary probable PTSD outcomes (Supplementary Table S6). Inflammation was unassociated with probable PTSD in females but was associated with elevated risk for clinically significant PTSD symptoms in males for certain markers (Supplementary Table S7). When adjusting for sociodemographic factors, inflammatory index levels were associated with each symptom cluster to a relatively similar extent among the full sample (Supplementary Table S8). Like overall PTSD symptoms, associations between inflammation and PTSD symptom clusters were stronger in males versus females, with the inflammatory index associated with each symptom cluster (especially negative alteration symptoms) in males (Supplementary Table S9).

## Discussion

Among individuals with recent trauma exposure, higher levels of plasma inflammatory cytokines measured in the ED were associated with higher levels of PTSD symptoms across 6 months. Effects differed by sex; among males, higher pro-inflammatory cytokines (the inflammatory index and IL-6, IL-8, and TNF-α) were associated with higher PTSD symptoms, whereas among females, higher IL-10 (which is generally considered anti-inflammatory) was associated with higher PTSD symptoms. Notably, associations we found were small in magnitude relative to some prior work (e.g. IL-8-PTSD symptom *β* = 0.39 [Cohen et al., 2011] and proinflammatory index-PTSD symptoms *r* = 0.26 [von Känel et al., 2022]), but we did still identify significant, covariate-adjusted associations in our larger, more diverse sample. With multiple repeated measures of PTSD symptoms across 6 months post-trauma, we did not find associations between ED levels of inflammation and changes in PTSD symptoms over time (e.g. decreasing symptoms or later onset); rather, inflammation was associated with higher PTSD symptoms on average across the full follow-up period. These findings suggest that inflammation is associated with overall PTSD severity (particularly among males), and this association is stable over time or does not impact trajectories of symptom change. Future work examining inflammation with different PTSD symptom trajectories and over longer periods of follow-up may provide further insight into the dynamics of these psychobiological processes.

Our findings are consistent with some prior work in smaller samples focused on peripheral inflammation. One study found that higher IL-8 during hospitalization was associated with higher post-traumatic stress symptoms 1 month later; the sample was majority male (71%), though sex differences were not examined (Cohen et al., 2011). Another study showed that an inflammatory index of IL-1β, IL-6, and TNF-α in the acute treatment phase following myocardial infarction was associated with higher PTSD symptoms over 12 months (von Känel et al., 2022). These prior findings were driven by IL-1β, which we did not measure, and sex differences were also not examined. In contrast, previous analyses identified that lower inflammation (especially TNF-α and IFN-γ) was predictive of chronic PTSD across a year (Michopoulos et al., 2020). Interestingly, among males in unadjusted models, lower IFN-γ was marginally associated with higher PTSD symptoms in our sample, though the association was imprecise and was nonsignificant once adjusted for potential confounders.

Peripheral inflammation, either induced directly by traumatic or chronic stress or stemming from individual genetic or predisposition differences, may impact brain functioning via active or leaky transport into the central nervous system or peripheral activation of neural pathways (Hori & Kim, 2019; Welcome, 2020). In turn, neuroinflammation may influence the development of PTSD symptoms through changes in plasticity, neurogenesis, and learning and memory (Hori & Kim, 2019). Interestingly, recent work using positron emission tomography in living people and transcriptomics in post-mortem brain tissue has identified a suppressed neuroimmune response in people with PTSD compared to controls (Bhatt et al., 2020; Bonomi et al., 2024; Hwang et al., 2025), even when peripheral inflammation indexed by CRP is elevated (Bhatt et al., 2020). Together with our current results and a vast literature showing elevated inflammatory activity following psychological or physical stress (Marsland et al., 2017), one potential model is that increased inflammatory activity in the immediate aftermath of traumatic stress could drive a pattern of immune signaling that diminishes microglial cell activation over time. Thus, inflammatory mechanisms of PTSD appear more complex than elevated peripheral proinflammatory cytokines driving neuroinflammation, warranting future research to better understand potential pathways linking both peripheral inflammation and neuroinflammation in the development of PTSD and progression over the course of PTSD. Better delineation of these relationships over time following trauma exposure could elucidate a more dynamic trajectory of peripheral inflammation and neuroimmune involvement. Although the current study cannot show causal effects or mechanistic pathways of peripheral inflammation on PTSD symptom development, findings suggest that heightened inflammation shortly after traumatic stress is associated with higher PTSD symptoms over time.

We found that generally pro-inflammatory markers were associated with subsequent PTSD symptoms, largely in males, indicating potential sex differences in the relationship between immune functioning and PTSD sequelae. Past work in the AURORA study has shown sex differences in PTSD symptom severity and in risk factors for PTSD development (including levels of acute dissociation, peritraumatic distress and anxiety, chance of dying during the trauma, and acute stress disorder) (Haering et al., 2024). Our findings suggest that post-trauma inflammation may be another important differential risk factor for PTSD between males and females. Stronger associations in males may be influenced by sex-related biological differences, including the influence of stress hormones on inflammatory response (Oertelt-Prigione, 2012). Females tend to exhibit higher inflammatory reactivity to acute stress and higher basal inflammation (Marsland et al., 2017; Oertelt-Prigione, 2012), though in our sample, ED inflammation was marginally higher in males than in females. Interestingly, we found that IL-10, considered an immune modulator with net anti-inflammatory effects, was associated with subsequent PTSD symptoms only in females, not males. IL-10 was positively correlated with pro-inflammatory cytokines in our sample, though it is unclear why IL-10 would be associated with PTSD in females but not males. In previous studies, IL-10 measured post-trauma was unassociated with PTSD up to a year later, though sex differences were not examined (de Oliveira et al., 2018; Michopoulos et al., 2020; Vedantam et al., 2021). It is possible that the sex differences we found were due in part to hormonal differences shown to influence inflammation (with estrogen and progesterone having anti-inflammatory effects on average) (King & Critchley, 2010; Oertelt-Prigione, 2012). While our current analyses were unable to incorporate menstrual cycle fluctuations in females or sex hormone levels in males or females, these factors should be considered when estimating sex differences in trauma, immune function, and PTSD symptom associations. Recent genetic modeling suggests that unique environmental effects influence sex differences in PTSD, and that genetic influences are stronger among females than among males (Amstadter et al., 2024). It is possible that other genetic and environmental factors exerted a stronger influence, relative to post-trauma inflammation, on PTSD sequelae in females, while inflammation was a more important relative influence in males. Interestingly, a prior study identified a differential sex effect between ED inflammation and PTSD over 12 months, with men having higher inflammation, which was associated with lower PTSD risk (Lalonde et al., 2021), an opposite direction from our findings. The reasons behind divergence in findings are unclear, though methodology differed for study and analytic design (e.g. the prior study drew blood very shortly [~3 hours] post-trauma and tested moderated mediation with pro-inflammatory cytokines as a mediator of the sex-PTSD relationship, moderation by steroid hormones [Lalonde et al., 2021]), and cytokine assay (i.e. the prior study used a multiplex Luminex assay, which may have lower reliability) (Chowdhury et al., 2009).

There were several limitations to the current study. Several factors that could have influenced inflammation were not adequately accounted for, including more traumatic stressor characteristics, ED and treatment-related factors, and preexisting and concurrent health status. In particular, the severity of trauma or physical injury are important potential confounder between inflammation and PTSD symptoms. However, all study participants had experiences that warranted ED visits; we adjusted for pain and NSAID use in the ED, and we adjusted for trauma type to try to mitigate this confounding concern. Moreover, it is possible that elevated absolute levels of inflammation following trauma increase risk for PTSD symptoms, regardless of the source of inflammation. Despite this, more severe trauma exposure/injuries may have been associated with higher inflammation in the ED and higher likelihood of PTSD symptoms, so residual confounding may be inflating our identified associations. Additionally, preexisting health factors (e.g. chronic inflammatory conditions) and medication use (e.g. anti-inflammatories and immunosuppressants) were unassessed but likely influenced ED inflammation and potentially could impact risk for PTSD symptom development, reflecting another important set of unadjusted confounders that might have biased associations up or down. There was also missingness among covariates; we used multiple imputation for missing covariate information, though non-completely random missingness may have introduced bias. Our analyses relied on self-reported PTSD symptoms, as opposed to gold-standard clinician-administered diagnostic assessments, though the PCL-5 has shown high validity and reliability for PTSD symptomology (Bovin et al., 2016) and our focus was on the severity of symptoms rather than the presence or absence of a clinical diagnosis. Despite being socio-demographically diverse, the sample relied on individuals who presented to an ED after experiencing a traumatic stressor in participating study sites; thus, our findings may not be representative of adults nationwide or of all individuals who experience trauma. Moreover, most traumatic experiences in the sample were motor vehicle collisions (74.8%), so findings may not accurately reflect post-traumatic sequelae due to other types of traumatic stress. Finally, we did not specifically involve people with lived experience in the study design, analyses, or interpretation, which could have enriched the impact of our work.

The current analyses benefitted from a large, diverse sample of individuals post-trauma with information on levels of inflammation in the close aftermath of trauma and repeated assessments of post-traumatic stress across time. We identified that peripheral inflammation assessed in the ED following trauma may be an important predictor of the development of PTSD, independent of other known risk and protective factors. This may have implications for PTSD risk prediction, whereby inflammation levels could feasibly be collected in the ED in conjunction with additional information for prediction models that identify individuals at highest risk for PTSD; these models could be used to allocate early intervention or prevention resources. Given recent technological advancements, there is great opportunity to integrate biomarkers into machine learning techniques for risk prediction or treatment selection in trauma-exposed individuals (Chekroud et al., 2021; Ramos-Lima et al., 2020). Moreover, the observed positive associations between inflammatory cytokines shortly after trauma and PTSD symptoms can inform additional mechanistic work focused on understanding psychobiological pathways to PTSD, determining causality of associations, and potentially contributing to the development of novel interventions (e.g. anti-inflammatory interventions) for PTSD. Additionally, our current findings suggest that inflammation may be predictive of post-traumatic sequelae in a sex-dependent manner, indicating possible hormonal correlates or influences that should be examined in future work.

## Supporting information

10.1017/S0033291726103833.sm001 Nishimi et al. supplementary material Nishimi et al. supplementary material
