# Supplementary material for: Inflammatory markers in the emergency department and PTSD symptoms in the AURORA Study: A longitudinal cohort study
Source: Psychol Med. 2026 May 8;56:e123. doi: 10.1017/S0033291726103833 (PMC13161806; doi:10.1017/S0033291726103833)
Supplement: Nishimi et al. supplementary material [file S0033291726103833sup001.docx]

**SUPPLEMENTAL TABLES**

| **Table S1. Covariates among the analytic sample versus excluded from the AURORA Cohort** | | | | |
| --- | --- | --- | --- | --- |
|  | | **Analytic Sample (n=742)** | **Excluded (n=2 201)** |  |
| **Continuous Covariates** | | *M (SD)* | *M (SD)* | *p-value* |
| Age | | 37.99 (13.7) | 35.20 (13.1) | **<.001** |
| State Area Deprivation Index | | 9.07 (3.4) | 9.37 (3.3) | **0.04** |
| ED Pain Level | | 6.38 (2.6) | 6.40 (2.7) | 0.88 |
| BMI | | 31.41 (8.7) | 29.32 (4.1) | 0.63 |
| Childhood Trauma Questionnaire | | 17.40 (15.7) | 17.23 (15.1) | 0.81 |
| Lifetime Trauma Count | | 3.31 (2.8) | 3.03 (2.8) | **0.02** |
| **Categorical Covariates** | | *N (%)* | *N (%)* | *p-value* |
| Sex | Female | 479 (64.6) | 1 339 (60.8) | 0.17 |
|  | Male | 263 (35.4) | 861 (39.1) |  |
| Race/Ethnicity | Non-Hispanic White | 255 (34.4) | 765 (34.8) | 0.50 |
|  | Non-Hispanic Black | 382 (51.5) | 1 076 (48.9) |  |
|  | Hispanic | 75 (10.1) | 267 (12.1) |  |
|  | Non-Hispanic Other | 26 (3.5) | 85 (3.9) |  |
| Education | Less than High School | 83 (11.2) | 256 (11.6) | 0.35 |
|  | High School Graduate | 180 (24.3) | 598 (27.2) |  |
|  | Some College | 205 (27.6) | 597 (27.1) |  |
|  | College Graduate | 273 (36.8) | 742 (33.7) |  |
| Family Income | Less than $19k/month | 236 (31.8) | 614 (27.9) | **<.001** |
|  | $19-35k/month | 220 (29.6) | 574 (26.1) |  |
|  | More than $35k/month | 239 (32.2) | 698 (31.7) |  |
| Employment | Employed Currently | 514 (69.3) | 1 388 (63.1) | **<.001** |
|  | Not Employed Currently | 184 (24.8) | 509 (23.1) |  |
| Marital Status | Married | 149 (20.1) | 459 (20.9) | 0.49 |
|  | Previously Married | 141 (19.0) | 366 (16.6) |  |
|  | Never Married | 447 (60.2) | 1 364 (62.0) |  |
| Trauma Type | Animal-related | 23 (3.1) | 40 (1.8) | 0.37 |
|  | Burns | 5 (0.7) | 9 (0.4) |  |
|  | Falls, <10 feet | 30 (4.0) | 132 (6.0) |  |
|  | Falls, ≥10 feet | 11 (1.5) | 40 (1.8) |  |
|  | Incident with many people | 2 (0.3) | 10 (0.5) |  |
|  | Motor Vehicle Collision | 555 (74.8) | 1 639 (74.5) |  |
|  | Non-Motorized Collision | 15 (2.0) | 38 (1.7) |  |
|  | Physical Assault | 69 (9.3) | 202 (9.2) |  |
|  | Sexual Assault | 4 (0.5) | 213 (0.6) |  |
|  | Other | 28 (3.8) | 78 (3.6) |  |
| ED NSAID Use | Yes | 254 (34.2) | 643 (29.2) | **0.04** |
|  | No | 349 (47.0) | 1 111 (50.5) |  |
| Alcohol Use | Yes | 437 (58.9) | 1 378 (62.6) | 0.14 |
|  | No | 303 (40.8) | 813 (36.9) |  |
| Tobacco Use | Yes | 235 (31.7) | 811 (36.8) | **0.04** |
|  | No | 503 (67.8) | 1 381 (62.7) |  |
| Pre-ED PTSD | PTSD | 340 (45.8) | 1 073 (48.8) | 0.18 |
|  | No PTSD | 402 (54.2) | 1 128 (51.2) |  |
| Pre-ED MDE | MDE | 172 (23.2) | 385 (17.5) | **<.001** |
|  | No MDE | 523 (70.5) | 1 313 (59.7) |  |
| *Note.* P-values are for ANOVAs or T-tests for covariates between analytic group versus excluded.  Previously married includes separated, annulled, divorced, or widowed; BMI = body mass index, ED = emergency department, MDE = major depressive episode, NSAID = non-steroidal anti-inflammatory drug, PTSD = posttraumatic stress disorder, SD = standard deviation  Missing: gender n=1 (0.0%), race/ethnicity n=12 (0.4%), education n=9 (0.3%), income n=362 (12.3%), employment n=348 (11.8%), marital status n=17 (0.6%), state area deprivation index n=33 (1.1%), ED NSAID use n=586 (19.9%), BMI n=2 545 (86.5%), alcohol use n=12 (0.4%), tobacco use n=13 (0.4%), childhood trauma questionnaire n=460 (15.6%), lifetime trauma count n=341 (11.6%), pre-ED MDE n=550 (18.7%) | | | | |

| **Table S2. ED inflammatory markers means and correlations (n=742)** | | | | | | | | |
| --- | --- | --- | --- | --- | --- | --- | --- | --- |
|  |  | **Raw** | **Log-Transformed** | 2. | 3. | 4. | 5. | 6. |
| **Inflammatory Marker** | *n* | *M (SD)* | *M (SD)* | *r* | *r* | *r* | *r* | *r* |
| 1. IL-6 | 737 | 3.24 (7.4) | 0.63 (0.9) | 0.24 | 0.18 | 0.06 | 0.24 | 0.58 |
| 2. IL-8 | 740 | 6.69 (5.8) | 1.68 (0.7) | -- | 0.22 | 0.10 | 0.16 | 0.61 |
| 3. TNF-α | 740 | 1.53 (2.1) | 0.34 (0.4) |  | -- | 0.44 | 0.55 | 0.72 |
| 4. IFN-γ | 739 | 7.22 (10.9) | 1.60 (0.8) |  |  | -- | 0.30 | 0.63 |
| 5. IL-10 | 727 | 0.47 (0.9) | -0.82 (0.6) |  |  |  | -- | 0.49 |
| 6. Inflammatory Index | 736 | -- | 0.01 (2.5) |  |  |  |  | -- |
| *r*s are Pearson correlations between log-transformed individual biomarkers and the inflammatory index (log-transformed IL-6, IL-8, TNF-α, and IFN-γ were standardized and summed) | | | | | | | | |

| **Table S3. PTSD symptoms over time (n=742)** | | | | | | | |
| --- | --- | --- | --- | --- | --- | --- | --- |
|  |  | **PTSD Symptoms** | **Probable PTSD** | 2 wks | 8 wks | 3 mos | 6 mos |
| **Timepoint** | *n* | *M (SD)* | *N (%)* | *r* | *r* | *r* | *r* |
| Pre-ED^*^ | 740 | 8.25 (5.6) | 340 (45.8) | 0.24 | 0.20 | 0.21 | 0.26 |
| 2 Weeks post-ED | 677 | 33.05 (19.8) | 317 (42.7) | -- | 0.29 | 0.29 | 0.22 |
| 8 Weeks post-ED | 688 | 31.08 (21.2) | 292 (39.4) |  | -- | 0.34 | 0.31 |
| 3 Months post-ED | 691 | 29.18 (21.5) | 263 (35.4) |  |  | -- | 0.39 |
| 6 Months post-ED | 698 | 27.29 (21.1) | 246 (33.2) |  |  |  | -- |
| *r*s are Pearson correlations between PTSD symptoms (PCL-5 total scores) over time; Probable PTSD is PCL-5 ≥33; ^*^Pre-ED PTSD is from the Brief PCL (potential range 0-24) and probable PTSD is Brief PCL ≥8 | | | | | | | |

| **Table S4. ED inflammatory markers and PTSD symptoms over time by sex** | | | | | |
| --- | --- | --- | --- | --- | --- |
|  | **Female (n=479)** | | **Male (n=263)** | |  |
|  | **Raw** | **Log-Transformed** | **Raw** | **Log-Transformed** | **Female vs Male** |
| **Inflammatory Marker** | *M (SD)* | *M (SD)* | *M (SD)* | *M (SD)* | *p-value* |
| IL-6 | 2.92 (6.8) | 0.58 (0.9) | 3.94 (8.2) | 0.71 (1.0) | *0.08* |
| IL-8 | 6.52 (5.8) | 1.65 (0.7) | 7.00 (5.8) | 1.75 (0.6) | *0.05* |
| TNF-α | 1.56 (2.5) | 0.34 (0.4) | 1.48 (0.8) | 0.36 (0.4) | 0.49 |
| IFN-γ | 7.74 (12.6) | 1.63 (0.8) | 6.27 (6.7) | 1.54 (0.7) | 0.13 |
| IL-10 | 0.48 (1.1) | -0.85 (0.6) | 0.46 (0.5) | -0.78 (0.6) | 0.11 |
| Inflammatory Index | -- | -0.07 (2.6) | -- | 0.15 (2.5) |  |
|  | **PTSD Symptoms** | **Probable PTSD** | **PTSD Symptoms** | **Probable PTSD** | **Female vs Male** |
| **PTSD Symptoms** | *M (SD)* | *N (%)* | *M (SD)* | *N (%)* | *p-value* |
| 2 Weeks post-ED | 34.04 (19.5) | 217 (45.3) | 31.17 (20.2) | 100 (38.0) | *0.07* |
| 8 Weeks post-ED | 31.60 (20.9) | 200 (41.8) | 30.14 (21.6) | 92 (35.0) | 0.39 |
| 3 Months post-ED | 30.48 (21.2) | 179 (27.4) | 26.71 (21.8) | 84 (31.9) | **0.03** |
| 6 Months post-ED | 28.92 (21.0) | 170 (35.5) | 24.18 (21.0) | 76 (28.9) | **0.01** |
| Inflammatory index (log-transformed IL-6, IL-8, TNF-α, and IFN-γ were standardized and summed); PTSD symptoms are PCL-5 total scores at each follow-up timepoint, and probable PTSD is PCL-5 ≥33.  Female vs male p-values are for T-tests comparing log-transformed inflammatory marker or PTSD symptoms | | | | | |

| **Table S5.** *Associations between inflammatory markers and repeated measures of posttraumatic stress symptoms over 6 months (n=742); inflammation*time and inflammation*sex interactions* | | | | | | | |
| --- | --- | --- | --- | --- | --- | --- | --- |
|  | **Time Interaction Models** | | |  | **Sex Interaction Models** | | |
| **Predictor** | *β* | *95% CI* | *p-value* | **Predictor** | *β* | *95% CI* | *p-value* |
| Inflammatory Index | 0.01 | -0.05, 0.08 | 0.684 | Inflammatory Index | **0.08** | **0.01, 0.14** | **0.026** |
| Time | 0.000 | -0.01, 0.004 | 0.881 | Sex (female) | **0.18** | **0.10, 0.26** | **<.001** |
| Inflam. Index*Time | 0.001 | -0.003, 0.01 | 0.566 | Inflam. Index*Sex (female) | *-0.07* | *-0.15, 0.01* | *0.100* |
|  |  |  |  |  |  |  |  |
| IL-6 | 0.03 | -0.03, 0.10 | 0.314 | IL-6 | **0.08** | **0.02, 0.14** | **0.011** |
| Time | 0.000 | -0.01, 0.004 | 0.896 | Sex (female) | **0.18** | **0.10, 0.26** | **<.001** |
| IL-6*Time | -0.001 | -0.01, 0.004 | 0.823 | IL-6*Sex (female) | **-0.09** | **-0.17, -0.01** | **0.034** |
|  |  |  |  |  |  |  |  |
| IL-8 | 0.04 | -0.03, 0.10 | 0.286 | IL-8 | **0.13** | **0.07, 0.19** | **<.001** |
| Time | 0.000 | -0.01, 0.004 | 0.876 | Sex (female) | **0.19** | **0.11, 0.27** | **<.001** |
| IL-8*Time | 0.002 | -0.003, 0.01 | 0.374 | IL-8*Sex (female) | **-0.10** | **-0.18, -0.03** | **0.010** |
|  |  |  |  |  |  |  |  |
| TNF-α | -0.02 | -0.08, 0.05 | 0.628 | TNF-α | 0.02 | -0.05, 0.08 | 0.639 |
| Time | 0.000 | -0.01, 0.004 | 0.880 | Sex (female) | **0.18** | **0.10, 0.26** | **<.001** |
| TNF-α*Time | 0.001 | -0.003, 0.01 | 0.583 | TNF-α*Sex (female) | -0.03 | -0.10, 0.05 | 0.509 |
|  |  |  |  |  |  |  |  |
| IFN-γ | -0.02 | -0.09, 0.05 | 0.597 | IFN-γ | -0.05 | -0.12, 0.01 | 0.121 |
| Time | 0.000 | -0.01, 0.004 | 0.888 | Sex (female) | **0.18** | **0.10, 0.26** | **<.001** |
| IFN-γ*Time | 0.001 | -0.004, 0.01 | 0.776 | IFN-γ*Sex (female) | 0.06 | -0.02, 0.14 | 0.156 |
|  |  |  |  |  |  |  |  |
| IL-10 | **0.07** | **0.003, 0.14** | **0.041** | IL-10 | 0.02 | -0.04, 0.09 | 0.465 |
| Time | 0.000 | -0.01, 0.004 | 0.889 | Sex (female) | **0.18** | **0.10, 0.26** | **<.001** |
| IL-10*Time | -0.003 | -0.01, 0.002 | 0.220 | IL-10*Sex (female) | 0.02 | -0.06, 0.10 | 0.588 |
| *Note.* Individual repeated measures linear regressions; missingness addressed by multiple imputation and presenting pooled results from 25 datasets. Inflammatory index is standardized sum of log IL-6, IL-8, TNF-α, and IFN-γ. All inflammatory predictors and PTSD symptoms are standardized. **Bold p<.05**; *Italicized p<.10.*  Covariates are time since ED, age, sex, race/ethnicity, education, income, employment, marital status, state area deprivation index, time from trauma to blood draw, site ID, and assay batch | | | | | | | |

**Figure S1. Unadjusted association between ED inflammatory index and averaged PTSD symptoms over 6 months, stratified by sex**


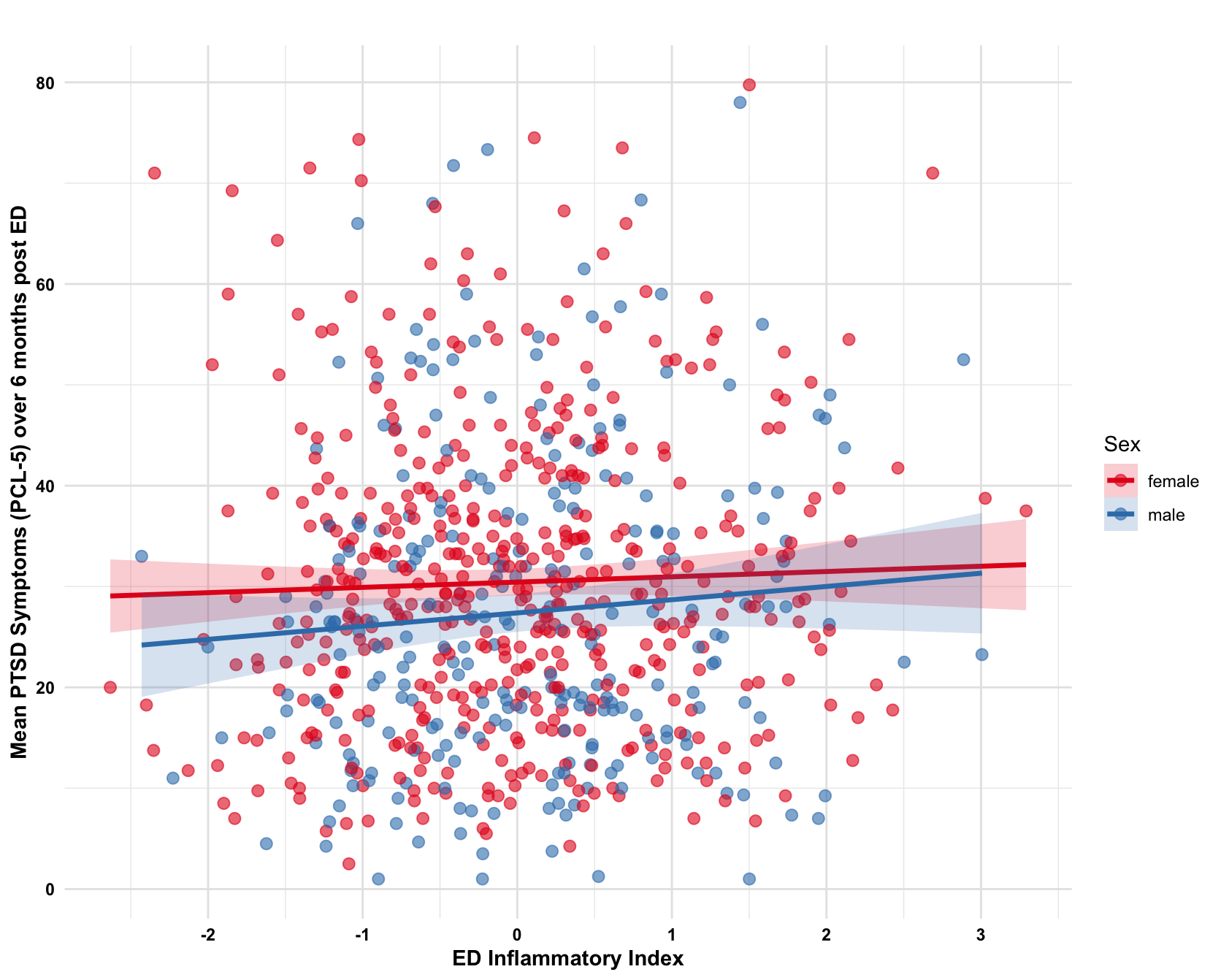


*Note*. Scatterplot of unadjusted associations between level of inflammatory index (standardized sum of log IL-6, IL-8, TNF-α, and IFN-γ levels) from the ED and average PTSD symptoms across 2 weeks, 8 weeks, 3 months, and 6 months of follow-up, stratified by sex (female = red, male = blue). Simple linear regression was used to create the line of best fit for each sex separately, surrounded by 95% confidence intervals. This visualizes the unadjusted association between ED inflammatory index and unstandardized average PTSD symptoms over follow-up; our primary models (Tables 2 and 3 in the main manuscript) reflect repeated regressions which account for time in a repeated measures framework and adjust for relevant covariates.

| **Table S6. Associations between inflammatory markers and repeated measures of probable PTSD over 6 months (n=742)** | | | | | | | | | |
| --- | --- | --- | --- | --- | --- | --- | --- | --- | --- |
|  | **Model 1** | | | **Model 2** | | | **Model 3** | | |
| **Predictor** | *RR* | *95% CI* | *p-value* | *RR* | *95% CI* | *p-value* | *RR* | *95% CI* | *p-value* |
| Inflammatory Index | 1.03 | 0.99, 1.08 | 0.155 | 1.02 | 0.98, 1.07 | 0.316 | 1.02 | 0.97, 1.07 | 0.384 |
| IL-6 | *1.04* | *1.00, 1.09* | *0.081* | *1.04* | *1.00, 1.09* | *0.066* | **1.06** | **1.01, 1.10** | **0.018** |
| IL-8 | **1.05** | **1.00, 1.10** | **0.038** | *1.04* | *0.99, 1.09* | *0.088* | 1.01 | 0.96, 1.05 | 0.804 |
| TNF-α | 1.01 | 0.97, 1.06 | 0.527 | 1.00 | 0.96, 1.05 | 0.925 | 1.00 | 0.95, 1.05 | 0.936 |
| IFN-γ | 0.98 | 0.94, 1.03 | 0.402 | 0.97 | 0.93, 1.02 | 0.265 | 0.99 | 0.94, 1.03 | 0.605 |
| IL-10 | 1.02 | 0.97, 1.06 | 0.418 | 1.03 | 0.98, 1.08 | 0.197 | 1.02 | 0.98, 1.07 | 0.367 |
| *Note.* Individual repeated measures Poisson regressions; missingness addressed by multiple imputation and presenting pooled results from 25 datasets. Inflammatory index is standardized sum of log IL-6, IL-8, TNF-α, and IFN-γ. All inflammatory predictors are standardized. Probable PTSD is PCL-5 scores ≥33. **Bold p<.05**; *Italicized p<.10.*  Model 1: time since ED  Model 2: time since ED, age, sex, race/ethnicity, education, income, employment, marital status, state area deprivation index, time from trauma to blood draw, site ID, and assay batch  Model 3: Model 1 and ED trauma type, ED pain level, ED NSAID use, childhood trauma questionnaire, lifetime trauma count, pre-ED PTSD, pre-ED depression, BMI, alcohol use, and tobacco use | | | | | | | | | |

| **Table S7. Sex-stratified associations between inflammatory markers and repeated measures of probable PTSD over 6 months** | | | | | | | | | |
| --- | --- | --- | --- | --- | --- | --- | --- | --- | --- |
|  | **Model 1** | | | **Model 2** | | | **Model 3** | | |
| **Predictor** | *RR* | *95% CI* | *p-value* | *RR* | *95% CI* | *p-value* | *RR* | *95% CI* | *p-value* |
| **Female (n=479)** |  |  |  |  |  |  |  |  |  |
| Inflammatory Index | 1.02 | 0.96, 1.07 | 0.503 | 0.99 | 0.94, 1.05 | 0.690 | 0.99 | 0.93, 1.05 | 0.665 |
| IL-6 | 1.02 | 0.97, 1.08 | 0.371 | 1.01 | 0.96, 1.07 | 0.734 | 1.03 | 0.97, 1.09 | 0.373 |
| IL-8 | 1.02 | 0.96, 1.07 | 0.574 | 1.00 | 0.95, 1.06 | 0.980 | 0.96 | 0.91, 1.02 | 0.193 |
| TNF-α | 0.99 | 0.94, 1.05 | 0.809 | 0.96 | 0.91, 1.02 | 0.161 | 0.97 | 0.91, 1.02 | 0.248 |
| IFN-γ | 1.01 | 0.96, 1.07 | 0.594 | 1.00 | 0.95, 1.06 | 0.979 | 1.01 | 0.96, 1.07 | 0.682 |
| IL-10 | 1.03 | 0.98, 1.09 | 0.217 | 1.02 | 0.97, 1.08 | 0.391 | 1.02 | 0.96, 1.07 | 0.579 |
|  |  |  |  |  |  |  |  |  |  |
| **Male (n=263)** |  |  |  |  |  |  |  |  |  |
| Inflammatory Index | *1.07* | *0.99, 1.16* | *0.089* | 1.06 | 0.98, 1.16 | 0.166 | 1.05 | 0.96, 1.15 | 0.319 |
| IL-6 | *1.08* | *1.00, 1.17* | *0.060* | *1.08* | *0.99, 1.18* | *0.081* | 1.07 | 0.99, 1.17 | 0.105 |
| IL-8 | **1.14** | **1.05, 1.23** | **0.001** | **1.11** | **1.02, 1.21** | **0.015** | 1.06 | 0.97, 1.16 | 0.198 |
| TNF-α | 1.06 | 0.98, 1.16 | 0.135 | 1.07 | 0.98, 1.16 | 0.139 | 1.07 | 0.97, 1.18 | 0.202 |
| IFN-γ | **0.90** | **0.83, 0.98** | **0.017** | **0.90** | **0.82, 0.99** | **0.028** | *0.92* | *0.83, 1.01* | *0.075* |
| IL-10 | 1.00 | 0.92, 1.09 | 0.986 | 1.00 | 0.92, 1.09 | 0.912 | 1.00 | 0.91, 1.09 | 0.965 |
| *Note.* Individual repeated measures linear regressions; missingness addressed by multiple imputation and presenting pooled results from 25 datasets. Inflammatory index is standardized sum of log IL-6, IL-8, TNF-α, and IFN-γ. All inflammatory predictors and PTSD symptoms are standardized. Probable PTSD is PCL-5 scores ≥33. **Bold p<.05**; *Italicized p<.10.*  Model 1: time since ED  Model 2: time since ED, age, race/ethnicity, education, income, employment, marital status, state area deprivation index, time from trauma to blood draw, and assay batch  Model 3: Model 1 and ED trauma type, ED pain level, ED NSAID use, childhood trauma questionnaire, lifetime trauma count, pre-ED PTSD, pre-ED depression, BMI, alcohol use, and tobacco use | | | | | | | | | |

| **Table S8. Associations between inflammatory markers and repeated measures of posttraumatic stress symptoms clusters over 6 months (n=742)** | | | | | | | | |
| --- | --- | --- | --- | --- | --- | --- | --- | --- |
|  | **Re-Experiencing** | | **Avoidance** | | **Negative Alterations** | | **Hyperarousal** | |
| **Predictor** | *β* | *95% CI* | *β* | *95% CI* | *β* | *95% CI* | *β* | *95% CI* |
| Inflammatory Index | **0.05** | **0.01, 0.09** | *0.04* | *-0.003, 0.08* | **0.06** | **0.02, 0.10** | **0.05** | **0.004, 0.09** |
| IL-6 | 0.01 | -0.03, 0.05 | 0.01 | -0.04, 0.05 | 0.02 | -0.02, 0.06 | 0.004 | -0.04, 0.05 |
| IL-8 | **0.11** | **0.07, 0.15** | **0.09** | **0.05, 0.13** | **0.09** | **0.05, 0.14** | **0.09** | **0.05, 0.13** |
| TNF-α | *0.04* | *-0.001, 0.08* | 0.03 | -0.01, 0.07 | **0.05** | **0.01, 0.09** | **0.05** | **0.01, 0.09** |
| IFN-γ | -0.03 | -0.07, 0.01 | -0.02 | -0.06, 0.02 | -0.01 | -0.05, 0.03 | -0.01 | -0.05, 0.03 |
| IL-10 | **0.05** | **0.01, 0.09** | **0.05** | **0.01, 0.09** | **0.10** | **0.06, 0.14** | **0.08** | **0.04, 0.12** |
| *Note.* Individual repeated measures linear regressions; missingness addressed by multiple imputation and presenting pooled results from 25 datasets. Inflammatory index is standardized sum of log IL-6, IL-8, TNF-α, and IFN-γ. All inflammatory predictors and PTSD symptoms are standardized. **Bold p<.05**; *Italicized p<.10.*  Covariates: time since ED, age, sex, race/ethnicity, education, income, employment, marital status, state area deprivation index, time from trauma to blood draw, site ID, and assay batch | | | | | | | | |

| **Table S9. Sex-stratified associations between inflammatory markers and repeated measures of posttraumatic stress symptoms clusters over 6 months** | | | | | | | | |
| --- | --- | --- | --- | --- | --- | --- | --- | --- |
|  | **Re-Experiencing** | | **Avoidance** | | **Negative Alterations** | | **Hyperarousal** | |
| **Predictor** | *β* | *95% CI* | *β* | *95% CI* | *β* | *95% CI* | *β* | *95% CI* |
| **Female (n=479)** |  |  |  |  |  |  |  |  |
| Inflammatory Index | 0.01 | -0.04, 0.07 | -0.003 | -0.06, 0.05 | 0.003 | -0.05, 0.06 | 0.01 | -0.04, 0.07 |
| IL-6 | 0.01 | -0.04, 0.06 | -0.001 | -0.05, 0.05 | 0.01 | -0.04, 0.06 | 0.01 | -0.04, 0.06 |
| IL-8 | **0.08** | **0.03, 0.14** | **0.06** | **0.01, 0.11** | *0.05* | *-0.004, 0.11* | *0.05* | *-0.002, 0.11* |
| TNF-α | -0.003 | -0.05, 0.05 | -0.03 | -0.08, 0.02 | -0.01 | -0.07, 0.04 | 0.01 | -0.04, 0.06 |
| IFN-γ | *-0.05* | *-0.10, 0.01* | -0.03 | -0.08, 0.02 | -0.03 | -0.09, 0.02 | -0.04 | -0.09, 0.02 |
| IL-10 | **0.07** | **0.01, 0.12** | *0.05* | *-0.004, 0.11* | **0.10** | **0.05, 0.16** | **0.10** | **0.05, 0.15** |
|  |  |  |  |  |  |  |  |  |
| **Male (n=263)** |  |  |  |  |  |  |  |  |
| Inflammatory Index | **0.11** | **0.04, 0.18** | **0.11** | **0.04, 0.17** | **0.17** | **0.11, 0.24** | **0.13** | **0.06, 0.19** |
| IL-6 | 0.02 | -0.05, 0.09 | 0.01 | -0.06, 0.08 | 0.06 | -0.02, 0.13 | 0.01 | -0.06, 0.09 |
| IL-8 | **0.16** | **0.09, 0.24** | **0.14** | **0.06, 0.21** | **0.18** | **0.12, 0.25** | **0.19** | **0.11, 0.26** |
| TNF-α | **0.13** | **0.06, 0.20** | **0.14** | **0.07, 0.21** | **0.18** | **0.11, 0.26** | **0.13** | **0.06, 0.21** |
| IFN-γ | -0.01 | -0.07, 0.05 | 0.01 | -0.05, 0.07 | 0.04 | -0.03, 0.10 | 0.01 | -0.05, 0.08 |
| IL-10 | 0.01 | -0.06, 0.09 | 0.04 | -0.03, 0.11 | **0.10** | **0.03, 0.18** | 0.05 | -0.03, 0.12 |
| *Note.* Individual repeated measures linear regressions; missingness addressed by multiple imputation and presenting pooled results from 25 datasets. Inflammatory index is standardized sum of log IL-6, IL-8, TNF-α, and IFN-γ. All inflammatory predictors and PTSD symptoms are standardized. **Bold p<.05**; *Italicized p<.10.*  Covariates: time since ED, age, race/ethnicity, education, income, employment, marital status, state area deprivation index, time from trauma to blood draw, site ID, and assay batch | | | | | | | | |
